# Determinants and prognostic value of onset symptoms in multiple sclerosis

**DOI:** 10.1007/s00415-025-13536-9

**Published:** 2025-12-02

**Authors:** Anna Karin Hedström, Tomas Olsson, Lars Alfredsson

**Affiliations:** 1 Department of Clinical Neuroscience, Center for Molecular Medicine, Karolinska Institutet, L8, Karolinska Institutet, 171 76 Stockholm, Sweden; 2 Institute of Environmental Medicine, Karolinska Institutet, Stockholm, Sweden; 3 Centre for Occupational and Environmental Medicine, Region Stockholm, Stockholm, Sweden

**Keywords:** Multiple sclerosis, Onset-symptom determinants, Disability milestones

## Abstract

**Background:**

Multiple sclerosis (MS) has heterogeneous clinical presentations at onset. We aimed to investigate determinants of MS onset-symptom type, and the prognostic relevance of onset categories for long-term disability outcomes.

**Methods:**

We analyzed 1385 incident MS cases from a Swedish population-based case–control study. Onset symptoms were hierarchically categorized as pyramidal/motor, brainstem/cerebellar, visual, or sensory, with sensory used as the reference. Associations with sex, ancestry, age at onset, smoking and body mass index were assessed using multinomial logistic regression. Disability progression was analyzed with Kaplan–Meier curves and Cox proportional hazards models for time to EDSS 3, 4, and 6.

**Results:**

Male sex was associated with higher odds of motor (OR 1.54, 95% CI 1.07–2.20) and brainstem/cerebellar onset (OR 1.36, 95% CI 1.00–1.84). Non-Nordic ancestry was linked to motor onset (OR 1.75, 95% CI 1.21–2.51). Obesity was strongly associated with visual onset (OR 2.22, 95% CI 1.41–3.40), and smoking with motor and brainstem/cerebellar onset. Motor onset consistently predicted shorter times to EDSS milestones. Brainstem/cerebellar onset conferred increased risk of reaching EDSS 4. In supplementary analyses, multifocal onset was associated with faster progression, but this effect was largely driven by concomitant motor involvement.

**Conclusions:**

The first clinical manifestation of MS varies by demographic and lifestyle characteristics and carries prognostic value for subsequent disability progression.

**Supplementary Information:**

The online version contains supplementary material available at 10.1007/s00415-025-13536-9.

## Introduction

Multiple sclerosis (MS) is a chronic, immune-mediated disease of the central nervous system characterized by heterogeneous clinical presentations and a variable disease course. The initial symptom at onset has historically been regarded as a potential prognostic marker, with sensory or visual onset generally associated with a more favorable trajectory compared with motor or multifocal onset [1, 2]. The prognostic implications of infratentorial symptoms, however, remain less clear. Some studies have suggested that cerebellar onset is associated with particularly poor outcomes, whereas brainstem onset may be more favorable [3], while others have reported a worse prognosis across both domains [4].

In addition, comparatively little attention has been directed to the determinants of onset-symptom type itself. It remains unclear whether demographic and lifestyle factors influence the type of first clinical manifestation in MS, or whether onset categories simply reflect random variation in lesion localization. Lifestyle-related exposures such as smoking and obesity are established risk factors for developing MS [5] and have also been associated with faster disability accumulation and earlier attainment of disability milestones [6, 7]. Whether such factors also influence the type of initial symptoms, and thereby potentially shape the early clinical manifestation of MS, has not been thoroughly examined. Clarifying such associations could provide insights into mechanisms underlying disease heterogeneity and indicate whether lifestyle-related risk factors act beyond disease susceptibility and progression to affect the earliest clinical expression of MS.

Using incident cases from a Swedish population-based case–control study, we aimed to investigate whether demographic and lifestyle characteristics are associated with onset-symptom type. We also compared time to disability milestones across onset groups to further clarify the prognostic relevance of onset categories.

## Methods

This study is nested within the Swedish population-based case–control study Epidemiologic Investigation of MS (EIMS). In EIMS, 3567 individuals with incident MS were recruited from neurology departments nationwide, including all university hospitals, between April 2005 and December 2019. The response rate among eligible cases was 93%. All MS diagnoses were confirmed by neurologists according to the McDonald criteria [8, 9]. At baseline, participants completed a standardized questionnaire covering demographic factors, lifestyle, and environmental exposures. The design and data collection procedures have been published elsewhere [10].

For the present analysis, we included all EIMS participants with relapsing-onset MS recruited through the two neurology clinics at Karolinska University hospital in Stockholm (*n* = 1385). All participants provided written informed consent. The study was approved by the Regional Ethical Review Board at Karolinska Institutet and conducted in accordance with the 1964 Declaration of Helsinki and its later amendments.

### Onset symptom ascertainment and classification

Onset symptoms were ascertained through a systematic review of medical records by trained physicians using a predefined abstraction protocol. Symptoms were mapped to Expanded Disability Status Scale (EDSS) functional systems: visual, sensory, pyramidal/motor, brainstem, cerebellar, autonomic, and cognitive [11]. We classified onset symptoms using a hierarchical scheme. Participants were assigned to the motor group if any pyramidal symptom was present at onset; otherwise, to the brainstem/cerebellar group if any brainstem or cerebellar symptom occurred without motor involvement; otherwise to the visual group without motor or brainstem/cerebellar involvement; otherwise to the sensory group if sensory symptoms co-occurred only with cognitive or autonomic domains (eTable S1).

### Measures

Demographic and lifestyle factors were obtained from baseline questionnaires. Sex and year of birth were recorded at study entry, and age at onset was calculated as age in years at the first MS symptom. Ancestry was classified as Nordic or non-Nordic origin. Cumulative smoking exposure was quantified as pack-years at the time of first symptoms, with one pack-year equivalent to having smoked 20 cigarettes per day for 1 year. Smoking exposure was categorized as never smoking (0 pack-years), < 10 pack-years, or ≥ 10 pack-years, consistent with prior MS literature. Body mass index (BMI) at the time of diagnosis was calculated as weight in kilograms divided by height in meters squared. Obesity was defined as BMI > 30 kg/m^2^. Educational level was categorized as pre-secondary, secondary (upper-secondary/high school), or post-secondary (typically university).

Clinical data were retrieved from the Swedish MS register [12], used across all neurology departments in Sweden as part of routine clinical documentation. The register includes prospectively recorded information on medical treatment, disease activity, and disability status, including repeated assessments of EDSS.

Primary outcomes were time from disease onset to first attainment of EDSS 3, EDSS 4, and EDSS 6, which represent widely used and clinically meaningful milestones of irreversible disability accumulation in MS. Follow-up time was calculated from first MS symptom until the onset of the events of interest, drop-out, death, or end of follow-up, whichever occurred first. Individuals already at a threshold at onset contributed an event at time 0. Participants not reaching a threshold were right censored at their last clinical assessment.

### Statistical analysis

Baseline characteristics were summarized overall and by onset-symptom category (sensory, visual, motor, brainstem/cerebellar, and multifocal). Continuous variables were presented as means with standard deviations (SD), and categorical variables as counts and percentages. Group differences were assessed using Chi-square tests for categorical variables and analysis of variance (ANOVA) for continuous variables.

Associations between demographic and lifestyle factors and symptom-onset type were evaluated using multinomial logistic regression with sensory onset as the reference category. The following categorical predictors were simultaneously included: sex, ancestry, age at onset (above cohort median or not), smoking, and obesity. We also assessed trends by including age, pack-years, and BMI as continuous variables, with further adjustment for sex and ancestry. Odds ratios (ORs) with 95% confidence intervals (CIs) were reported for each non-reference onset category versus sensory, with continuous predictors expressed per one-unit increase.

For disability outcomes, we generated Kaplan–Meier curves by symptom-onset group for time to EDSS 3, 4, and 6. Group differences were assessed with the log-rank test. For presentation, survival plots were truncated at 20 years.

To quantify group differences, we fitted Cox proportional hazards models for time to EDSS 3, 4, and 6 with symptom-onset category as the main exposure. Hazard ratios (HR) with 95% CI were reported versus the sensory group. The proportional hazards assumption was tested using Schoenfeld residuals, with no violations of proportionality observed. Analyses controlled for sex, ancestry, age at diagnosis, educational attainment, pack-years of smoking, BMI, and treatment. Treatment exposure was modeled as a time-varying categorical covariate (untreated, platform therapy, or high-efficacy therapy), updated at treatment initiation and at class switches. All models additionally included calendar year of diagnosis to account for secular changes in diagnostic practices, treatment availability, and follow-up intensity.

Since brainstem and cerebellar symptoms may differ in prognostic value, we also examined the prognostic impact of brainstem onset separately by excluding all cases with cerebellar involvement in the brainstem/cerebellar group.

As a prespecified sensitivity analysis, we also applied a single- versus multifocal classification. Single-system onset comprised exclusively sensory, exclusively visual, exclusively motor, and single-system brainstem/cerebellar involvement. Multifocal onset was defined as concurrent involvement of two or more functional systems at onset. Cognitive or autonomic symptoms were not observed in isolation. When present with any other system they were counted as additional systems and therefore classified as multifocal (eTable S2).

In additional analyses, the multifocal group was divided into those with and without motor involvement at onset, in order to disentangle the effect of multifocality per se from the impact of motor symptoms.

Finally, for each endpoint (EDSS 3/4/6), we repeated the Cox models after excluding participants who had already reached the respective milestone at diagnosis.

We conducted a sensitivity analysis to examine educational attainment in relation to onset-symptom type, recognizing that education at diagnosis may not reflect final education among younger individuals. To mitigate age-related misclassification, this analysis was restricted to participants aged > 25 years and included education (pre-secondary, secondary, or post-secondary) as a covariate alongside the other predictors. All analyses were conducted in SAS 9.4 (SAS Institute Inc, Cary, NC, USA).

## Results

The most frequent initial presentation was sensory, followed by brainstem/cerebellar, visual, and motor symptoms. Age at onset was lowest in the visual group and highest in the brainstem/cerebellar group. Women accounted for 73% of the cohort, with the proportion highest among those with visual onset and lowest among those with motor onset. Non-Nordic ancestry was most common in the motor group. Median baseline EDSS was 2.0 (IQR 1.0–2.5) in the motor-onset group, 1.5 (1.0–2.0) in the brainstem/cerebellar group, 1.5 (1.0–2.5) in the visual group, and 1.5 (1.0–3.0) in the sensory group. Lifestyle factors also differed by onset type. Cumulative smoking exposure was greatest in the motor and brainstem/cerebellar groups, whereas obesity prevalence was highest among those with visual onset. Baseline characteristics by onset-symptom group are presented in Table 1.

**Table 1:** Characteristics of participants, overall and by symptom-onset type

	Total	Motor	Brainstem/cerebellar	Visual	Sensory	*p*-value
*n*	1385	194	334	318	539	
Age at disease onset (SD)	32.7 (10.0)	33.5 (10.2)	34.0 (10.8)	30.9 (9.3)	32.7 (9.7)	** < 0.0001**
Female, *n* (%)	1015 (73.3)	128 (66.0)	228 (68.3)	254 (79.9)	405 (75.1)	** < 0.0001**
Male, *n* (%)	370 (26.7)	66 (34.0)	106 (31.7)	64 (20.1)	134 (24.9)	
Nordic, *n* (%)	1026 (74.1)	129 (66.5)	245 (73.4)	235 (73.9)	417 (77.4)	**0.03**
Non-Nordic, *n* (%)	359 (25.9)	65 (33.5)	89 (26.6)	83 (26.1)	122 (22.6)	
Pre-secondary education, *n* (%)	102 (7.4)	29 (11.3)	29 (8.7)	21 (6.6)	30 (5.6)	0.09
Secondary education, *n* (%)	662 (44.9)	79 (40.7)	151 (45.2)	160 (50.3)	232 (43.0)	
Post-secondary education, *n* (%)	661 (47.7)	93 (47.9)	154 (46.1)	137 (43.1)	277 (51.4)	
Time to diagnosis (SD)	2.7 (3.6)	3.0 (3.9)	2.5 (3.7)	2.7 (3.8)	2.7 (3.3)	0.07
DMT, *n* (%)	1329 (96.0)	190 (97.9)	320 (95.8)	305 (95.9)	514 (95.4)	0.48
Time to DMT (SD)	3.9 (4.8)	4.6 (5.1)	3.5 (4.2)	4.0 (5.3)	4.0 (4.7)	0.08
Proportion of follow-up on DMT (SD)	0.9 (0.2)	0.9 (0.2)	0.9 (0.2)	0.9 (0.2)	0.9 (0.2)	0.27
First recorded EDSS, mean (SD)	1.9 (1.4)	2.3 (1.6)	1.9 (1.5)	1.7 (1.3)	1.9 (1.4)	** < 0.0001**
First recorded EDSS, median (IQR)	2.0 (1.0–2.5)	2.0 (1.0–2.5)	1.5 (1.0–2.0)	1.5 (1.0–2.5)	1.5 (1.0–3.0)	
Never smoker	631 (45.6)	85 (43.8)	136 (40.7)	146 (45.9)	264 (49.0)	**0.02**
> 0, < 10 pack-years	529 (38.2)	69 (35.6)	130 (38.9)	130 (40.9)	200 (37.1)	
> 10 pack-years	225 (16.3)	40 (20.6)	68 (20.4)	42 (13.2)	75 (13.9)	
Pack-years (SD)	7.0 (8.7)	8.1 (8.5)	8.7 (9.9)	5.8 (7.1)	7.0 (9.1)	**0.002**
Mean BMI	24.4 (4.7)	24.4 (4.3)	24.4 (4.6)	24.6 (4.7)	24.2 (4.9)	0.65
Obesity; BMI > 30, *n* (%)	148 (10.7)	17 (8.8)	36 (10.8)	49 (15.4)	46 (8.5)	**0.01**

Bold p-values indicate significant differences between onset groups (p < 0.05)

In multinomial logistic regression with sensory onset as the reference group, male sex was associated with a higher probability of motor (OR 1.54, 95% CI 1.07–2.20) and brainstem/cerebellar onset (OR 1.36, 95% CI 1.00–1.84). Non-Nordic ancestry was linked to motor onset (OR 1.75, 95% CI 1.21–2.51). Obesity showed a strong association with visual onset (OR 2.22, 95% CI 1.41–3.40), as did higher BMI as a continuous measure (OR per unit 1.03, 95% CI 1.00–1.06). In the model with continuous predictors, older age at onset was inversely associated with visual onset (OR per year 0.98, 95% CI 0.96–0.99), while cumulative smoking exposure was associated with motor (OR 1.05, 95% CI 1.00–1.10) and brainstem/cerebellar onset (OR 1.06, 95% CI 1.01–1.11) (Table 2).

**Table 2:** Demographic and lifestyle factors associated with onset-symptom type (hierarchical classification; reference: sensory)

Predictor	OR (95% CI)	OR (95% CI)^a^
Motor onset (*n* = 194)		
Male	**1.54 (1.07–2.20)**	
Non-Nordic	**1.74 (1.21–2.51)**	
> Median age at onset	1.23 (0.87–1.74)	1.01 (0.99–1.02)
1–10 pack-years of smoking	1.14 (0.78–1.65)	**1.05 (1.00–1.10)**
> 10 pack-years of smoking	**1.59 (1.01–2.32)**	
BMI	1.01 (0.77–1.97)	1.00 (0.96–1.04)
Brainstem/cerebellar (*n* = 334)		
Male	**1.36 (1.00–1.84)**	
Non-Nordic	1.33 (0.82–2.18)	
Age at onset	1.04 (0.78–1.39)	1.01 (0.99–1.02)
1–10 pack-years of smoking	1.85 (0.94–1.74)	**1.06 (1.01–1.11)**
> 10 pack-years of smoking	**1.72 (1.15–2.37)**	
BMI	1.23 (0.80–1.97)	1.00 (0.97–1.03)
Visual (*n* = 318)		
Male	0.74 (0.53–1.05)	
Non-Nordic	1.12 (0.81–1.55)	
Age at onset	0.82 (0.61–1.09)	**0.98 (0.96–0.99)**
1–10 pack-years of smoking	1.09 (0.88–1.60)	0.99 (0.94–1.05)
> 10 pack-years of smoking	1.05 (0.67–1.54)	
BMI	**2.22 (1.41–3.40)**	**1.03 (1.00–1.05)**

Bold values indicate significant associations (p < 0.05)

Sensory onset, co-occurring only with cognitive or autonomic domains, was used as the reference category (*n* = 539). All variables were entered simultaneously in the model

^a^For continuous predictors, estimates correspond to a 1-unit increase (years, pack-years of smoking, BMI)

**Fig. 1:**
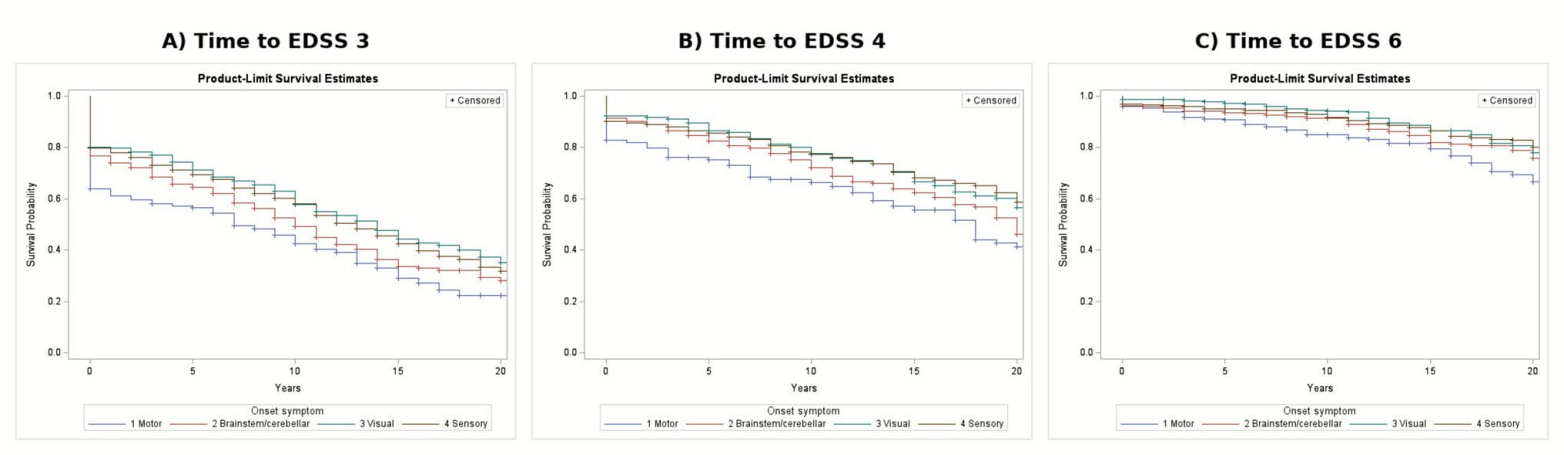
Kaplan–Meier survival curves for time to EDSS milestones from disease onset by symptom-onset group. **A** Time to EDSS 3 (log-rank *p* < 0.0001); **B** time to EDSS 4 (log-rank *p* < 0.0001); **C** time to EDSS 6 (log-rank *p* = 0.004). Participants not reaching the respective milestone were censored at last follow-up

Kaplan–Meier curves for time to EDSS milestones by onset-symptom group are shown in Fig. 1. Group differences were statistically significant for EDSS 3 (log-rank *p* < 0.0001), EDSS 4 (log-rank *p* < 0.0001) and EDSS 6 (*p* = 0.004).

Compared with sensory onset, participants with motor onset had a higher risk of reaching EDSS 3 (HR 1.38, 95% CI 1.13–1.69), EDSS 4 (HR 1.58, 95% CI 1.23–2.03), and EDSS 6 (HR 1.69, 95% CI 1.19–2.40). Participants with brainstem/cerebellar onset had an increased risk of reaching EDSS 4 (HR 1.23, 95% CI 1.00–1.54). Results were consistent in unadjusted and adjusted models (Table 3).

**Table 3:** Hazard ratio and 95% confidence interval for disability progression by onset-symptom (hierarchical) compared with the sensory onset group

	*N*	Time (SD)	Outcome (%)	HR (95% CI)^a^	HR (95% CI)^b^
EDSS 3					
Motor	194	7.6 (7.8)	141 (72.7)	**1.43 (1.17–1.74)**	**1.39 (1.14–1.71)**
Brainstem/cerebellar	334	8.2 (7.2)	204 (61.1)	1.17 (0.98–1.40)	1.12 (0.93–1.34)
Visual	318	9.5 (7.7)	168 (52.8)	0.92 (0.76–1.11)	0.94 (0.78–1.14)
Sensory	539	9.3 (7.3)	307 (57.0)	1.0 (reference)	1.0 (reference)
EDSS 4					
Motor	194	11.3 (8.3)	99 (51.0)	**1.66 (1.30–2.13)**	**1.58 (1.23–2.02)**
Brainstem/cerebellar	334	11.4 (7.5)	134 (40.1)	**1.32 (1.05–1.65)**	**1.23 (1.00–1.53)**
Visual	318	12.4 (7.7)	106 (33.3)	1.02 (0.80–1.30)	1.06 (0.83–1.35)
Sensory	539	12.3 (7.6)	176 (32.7)	1.0 (reference)	1.0 (reference)
EDSS 6					
Motor	194	14.6 (7.7)	53 (27.3)	**1.75 (1.24–2.47)**	**1.69 (1.19–2.40)**
Brainstem/cerebellar	334	13.7 (7.3)	62 (18.6)	1.28 (0.92–1.78)	1.21 (0.87–1.68)
Visual	318	14.5 (7.4)	47 (14.8)	0.97 (0.68–1.39)	1.06 (0.74–1.53)
Sensory	539	14.5 (7.3)	83 (15.4)	1.0 (reference)	1.0 (reference)

Bold HRs indicate significant associations with time to EDSS milestones (p < 0.05)

^a^Unadjusted

^b^Adjusted for age, sex, ancestry, calendar year of disease onset, educational attainment, smoking, BMI status, and DMT exposure

Excluding participants with cerebellar symptoms from the brainstem/cerebellar group did not materially change the results. For EDSS 4, the HR remained elevated at 1.32 (95% CI 1.03–1.69) compared to sensory onset.

In sensitivity analyses applying a single- versus multifocal classification of onset symptoms (eTable S2), associations between demographic and lifestyle factors and onset type, as well as prognostic outcomes, were consistent with the main analyses (eTables S3–S4). When stratifying the multifocal group by motor involvement, only participants with motor symptoms showed an increased risk of disability progression (EDSS 4: HR 1.53, 95% CI 1.13–2.08; EDSS 6: HR 1.86, 95% CI 1.23–2.81), whereas multifocal onset without motor involvement was not significantly associated with poorer outcomes (EDSS 4: HR 1.10, 95% CI 0.80–1.514; EDSS 6: HR 1.25, 95% CI 0.81–1.96).

In sensitivity analyses excluding individuals who had already reached the respective milestone at diagnosis, estimates for EDSS 4 and 6 were consistent with the main analyses. By contrast, for EDSS 3, between-group differences were attenuated and no longer significant (eTable S5).

In the age restricted analysis (> 25 years at diagnosis) including education, educational attainment was not significantly associated with onset–symptom type, while the associations for smoking and BMI remained consistent (eTable S6).

## Discussion

Our results suggest that both demographic and lifestyle factors influence the clinical presentation of MS at onset. Obesity was strongly linked to visual onset, while cumulative smoking exposure was associated with both motor and brainstem/cerebellar onset. Motor symptoms were consistently associated with shorter times to EDSS milestones compared with sensory onset. Brainstem/cerebellar onset predicted a higher risk of reaching EDSS 4, while the association with EDSS 6 was less pronounced. For multifocal onset, the increased risk of progression appeared largely driven by concomitant motor involvement, indicating that the prognostic impact of multifocal presentations may depend on which functional systems are affected.

In line with previous reports, male sex was associated with motor and brainstem/cerebellar onset, whereas women were more likely to present with sensory or visual symptoms [13, 14]. Younger age at onset was associated with visual onset, consistent with previous registry data showing that optic neuritis tends to present at younger ages than other MS onset types [2]. In addition, we found that non-Nordic ancestry was associated with an increased likelihood of motor onset. This is consistent with previous evidence that genetic ancestry may influence onset-symptom type [15, 16].

Smoking is a well-established risk factor for MS onset and progression [5], but its association with initial symptom type has been less well explored. Our findings suggest that cumulative smoking exposure increases the likelihood of motor, brainstem/cerebellar and multifocal involvement at disease onset. These findings may reflect the non-selective proinflammatory and neurotoxic effects of tobacco smoke, including oxidative stress, endothelial dysfunction, and impaired blood–brain barrier integrity [17]. Similar patterns were reported in a Swiss study [18], supporting an association between smoking exposure and the initial clinical presentation of MS.

Obesity, on the other hand, showed a strong association with visual onset, consistent with epidemiological studies linking obesity to a higher incidence of optic neuritis (ON) in the general population [19]. Beyond ON risk, several optical coherence tomography studies in non-MS populations suggest that the metabolic and inflammatory consequences of obesity may accelerate retinal neurodegeneration, including faster thinning of the retinal nerve fiber layer [19–22]. Importantly, similar findings have also been reported in MS, where higher BMI was associated with faster rates of retinal atrophy over time [23]. Mechanistically, obesity may contribute to optic nerve vulnerability through chronic low-grade inflammation, dysregulated adipokine signaling, and metabolic stress on oligodendrocytes and retinal ganglion cells [24, 25], which could lower the threshold for inflammatory demyelination in the visual pathway. Systemic factors such as leptin resistance, insulin dysregulation, and altered lipid metabolism have also been implicated in promoting proinflammatory Th17 responses that may amplify the susceptibility of the retinal/optic pathway [26–28]. Together, these observations suggest that obesity may influence not only MS susceptibility and progression but also shape the initial clinical presentation by predisposing to visual involvement.

Consistent with previous reports, we observed that motor onset was associated with a faster progression to EDSS milestones, while sensory and visual onset were associated with slower progression [29–31]. Differences were most pronounced for EDSS 4, which may partly reflect the construction of the EDSS scale, as it disproportionately emphasizes ambulation and pyramidal function. However, the poorer prognosis associated with motor onset likely also reflects greater axonal injury at onset.

Adjusted models indicated onset symptoms retained their prognostic value after accounting for demographic, lifestyle, and treatment factors, although some estimates were attenuated. This suggests that onset symptoms carry independent prognostic information, while lifestyle factors may contribute to, but not fully explain, these differences.

Our findings contribute to the understanding of the prognostic implications of infratentorial onset. Brainstem involvement was associated with a less favorable disability trajectory compared to sensory onset. Previous studies have reported mixed results [2–4, 32]. A large MSBase study described brainstem onset as relatively favorable [3], whereas a systematic review concluded that infratentorial onset in general predicts poorer outcomes [4]. Such discrepancies may partly reflect the differences in classification and reference categories.

A limitation of our study relates to how disability milestones were handled in survival analyses. Some participants had already reached a given EDSS threshold by the time of their first clinical assessment after symptom onset. In our main analyses these individuals were treated as having attained the milestone at time 0, which may underestimate the true time from onset to disability. Excluding such cases, however, would selectively remove individuals who had already reached the threshold and thereby overestimate time to event. Thus neither approach is ideal, with the potential for bias arising from diagnostic delay and pre-baseline disability accumulation. When we excluded individuals who had already reached a milestone at diagnosis, results for EDSS 4 and 6 remained consistent with the main analyses. For EDSS 3, the between-group differences were attenuated and lost statistical significance, suggesting that early attainment of EDSS 3 may partly account for the stronger signal in the main analysis and that EDSS 3 is more sensitive to diagnostic delay and pre-baseline disability. Onset categories reflect only the first symptom, whereas most patients subsequently develop relapses from other systems. Onset type should thus be regarded as an early marker rather than a phenotype. The EDSS scale favors detection of differences in ambulation, which may partly drive the associations with motor onset. Classification of onset symptoms relied on retrospective medical record review, which may introduce misclassification. BMI was measured at diagnosis rather than at symptom onset, which may introduce exposure misclassification if weight changed between onset and diagnosis. Our study was limited to patients recruited from two Stockholm clinics, which may reduce generalizability compared with the entire EIMS cohort, though the participating clinics serve large and diverse catchment areas. Some onset subgroups were relatively small, limiting statistical power and precision of estimates. Treatment exposure was simplified to first DMT category and time to initiation, though treatment in reality is time-varying and more complex. Finally, lifestyle variables were self-reported at diagnosis, without capturing post-diagnosis changes, raising the possibility of misclassification and residual confounding.

In conclusion, our findings indicate that onset symptoms reflect, at least in part, background exposures and demographic characteristics, and carry prognostic information for disability milestones. These findings support onset symptoms as meaningful clinical markers of disease trajectory, while also pointing to a contributory role of lifestyle factors in shaping the initial manifestation of MS.

## Supplementary Information

Below is the link to the electronic supplementary material.

Supplementary file 1 (DOCX 29 KB)

## Data Availability

Anonymized data underlying this article will be shared on reasonable request from any qualified investigator that wants to analyze questions that are related to the published article.
