# Supplementary material for: Determinants and prognostic value of onset symptoms in multiple sclerosis
Source: J Neurol. 2025 Dec 2;273(1):2. doi: 10.1007/s00415-025-13536-9 (PMC12672741; doi:10.1007/s00415-025-13536-9)
Supplement: Supplementary file 1 — (DOCX 29 KB) [file 415_2025_13536_MOESM1_ESM.docx]

eTable 1. Hierarchical classification of onset symptoms (N=1385)

|  | | Classification using hierarchical scheme | | | | |
| --- | --- | --- | --- | --- | --- | --- |
| Symptoms | | | | | Category | N (%) |
| Motor | Brainstem/cerebellar | | Visual | Sensory |  |  |
| + | -/+ | | -/+ | -/+ | Motor | 194 (14.0) |
| - | + | | -/+ | -/+ | Brainstem/cerebellar | 334 (24.1) |
| - | - | | + | -/+ | Visual | 318 (23.0) |
| - | - | | - | + | Sensory | 539 (38.9) |

Participants were assigned to the motor group if any pyramidal symptom was present at onset, otherwise to the brainstem/cerebellar group if any brainstem or cerebellar symptom occurred without motor involvement, otherwise to the visual group without motor or brainstem/cerebellar involvement, otherwise to the sensory group if sensory symptoms co-occurred only with cognitive or autonomic domains.

eTable 2. Single-multifocal classification of onset symptoms (N=1385)

| Single- versus multifocal classification | N (%) |
| --- | --- |
| Exclusively sensory | 539 (38.9) |
| Exclusively visual | 299 (21.6) |
| Single-system brainstem/cerebellar | 203 (14.7) |
| Exclusively pyramidal | 74 (5.3) |
| Multifocal | 270 (19.5) |

Single-system pyramidal/brainstem/cerebellar” indicates onset confined to one of these systems.

eTable 3. Demographic and lifestyle factors associated with onset symptom type (single-multifocal classification; reference: sensory)

| Visual onset (n=299) | | |
| --- | --- | --- |
| Predictor | OR (95% CI) | Trend |
| Male | 0.73 (0.54-1.08) |  |
| Non-Nordic | 1.15 (0.82-1.61) |  |
| >Median age at onset | 0.85 (0.63-1.14) | **0.98 (0.96-0.99)** |
| 1-10 pack years of smoking >10 pack years of smoking | 1.11 (0.70-1.76) 1.14 (0.72-1.79) | 1.00 (0.94-1.05) |
| BMI | **2.25 (1.44-3.51)** | **1.03 (1.00-1.06)** |
| Brainstem/cerebellar (n=203) | | |
| Male | **1.54 (1.08-2.20)** |  |
| Non-Nordic | 1.04 (0.70-1.53) |  |
| >Median age at onset | 1.15 (0.82-1.62) | 1.01 (1.00-1.03) |
| 1-10 pack years of smoking >10 pack years of smoking | 1.44 (1.00-1.58) **1.63 (1.01-2.62)** | **1.05 (1.00-1.11)** |
| BMI | 1.56 (0.93-2.60) | 1.01 (0.97-1.05) |
| Motor (n=74) | | |
| Male | **1.82 (1.09-3.04)** |  |
| Non-Nordic | **1.75 (1.03-2.97)** |  |
| >Median age at onset | 0.97 (0.57-1.63) | 1.00 (0.97-1.03) |
| 1-10 pack years of smoking >10 pack years of smoking | 0.94 (0.67-1.15) **2.08 (1.10-3.94)** | **1.11 (1.02-1.20)** |
| BMI | 1.45 (0.70-3.15) | 1.03 (0.98-1.02) |
| Multifocal (n=270) | | |
| Male | 1.18 (0.85-1.65) |  |
| Non-Nordic | **1.55 (1.11-2.16)** |  |
| >Median age at onset | 1.06 (0.78-1.45) | 1.00 (0.98-1.02) |
| 1-10 pack years of smoking >10 pack years of smoking | 1.26 (0.92-1.74) 1.49 (0.95-2.28) | 1.04 (0.98-1.09) |
| BMI | 0.87 (0.56-1.52) | 0.99 (0.96-1.02) |

Sensory onset was used as the reference category (n=539). All variables were entered simultaneously in the model. For continuous predictors, estimates correspond to a 1-unit increase (years, pack-years of smoking, BMI). motor

eTable 4. Disability progression by onset symptom (single-multifocal)

| EDSS 3 | | | | | |
| --- | --- | --- | --- | --- | --- |
|  | N | Time (SD) | Outcome (%) | HR (95% CI) | HR (95% CI)^1^ |
| Sensory | 539 | 9.3 (7.3) | 307 (57.0) | 1.0 (reference) | 1.0 (reference) |
| Visual | 299 | 9.5 (7.6) | 159 (53.2) | 0.92 (0.76-1.11) | 0.94 (0.78-1.14) |
| Brainstem/cerebellar | 203 | 8.1 (6.9) | 124 (61.1) | 1.19 (0.97-1.47) | 1.09 (0.88-1.35) |
| Motor | 74 | 7.8 (8.5) | 55 (74.3) | **1.42 (1.07-1.90)** | **1.34 (1.00-1.79)** |
| Multifocal | 270 | 8.1 (7.5) | 175 (64.8) | **1.24 (2.03-1.49)** | **1.23 (1.02-1.48)** |
| EDSS 4 | | | | | |
| Sensory | 539 | 12.3 (7.6) | 176 (32.7) | 1.0 (reference) | 1.0 (reference) |
| Visual | 299 | 12.4 (7.8) | 101 (33.8) | 1.03 (0.81-1.32) | 1.06 (0.83-1.36) |
| Brainstem/cerebellar | 203 | 11.5 (7.2) | 89 (43.8) | **1.44 (1.12-1.86)** | **1.28 (1.00-1.65)** |
| Motor | 74 | 11.8 (9.1) | 42 (56.8) | **1.74 (1.24-2.43)** | **1.56 (1.11-2.20)** |
| Multifocal | 270 | 11.2 (7.7) | 107 (39.6) | **1.32 (1.04-1.67)** | **1.30 (1.01-1.65)** |
| EDSS 6 | | | | | |
| Sensory | 539 | 14.5 (7.3) | 83 (15.4) | 1.0 (reference) | 1.0 (reference) |
| Visual | 299 | 14.4 (7.5) | 43 (14.4) | 0.95 (0.66-1.37) | 0.05 (0.72-1.52) |
| Brainstem/cerebellar | 203 | 13.9 (7.2) | 40 (19.7) | 1.34 (0.92-1.95) | 1.19 (0.81-1.76) |
| Motor | 74 | 15.9 (8.3) | 21 (28.4) | **1.62 (1.00-2.61)** | 1.50 (0.92-2.47) |
| Multifocal | 270 | 13.7 (7.3) | 58 (21.5) | **1.50 (1.07-2.09)** | **1.53 (1.09-2.15)** |

^1^Adjusted for age, sex, ancestry, calendar year of disease onset, educational attainment, smoking, BMI status, and DMT exposure.

eTable 5. Disability progression by onset symptom (hierarchical)

| EDSS 3 | | | | | |
| --- | --- | --- | --- | --- | --- |
|  | N | Time (SD) | Outcome (%) | HR (95% CI)^1^ | HR (95% CI)^2^ |
| Motor | 194 | 9.5 (7.0) | 74 (38.1) | 1.13 (0.87-1.48) | 1.11 (0.85-1.45) |
| Brainstem/cerebellar | 334 | 8.6 (6.8) | 40 (40.0) | 1.19 (0.95-1.48) | 1.14 (0.91-1.42) |
| Visual | 318 | 10.6 (7.2) | 105 (33.0) | 0.86 (0.68-1.09) | 0.88 (0.70-1.13) |
| Sensory | 539 | 10.5 (6.5) | 201 (37.3) | 1.0 (reference) | 1.0 (reference) |
| EDSS 4 | | | | | |
| Motor | 194 | 12.4 (7.3) | 67 (34.5) | **1.59 (1.18-2.14)** | **1.50 (1.11-2.03)** |
| Brainstem/cerebellar | 334 | 11.8 (7.0) | 29 (29.0) | **1.50 (1.16-1.95)** | **1.39 (1.07-1.81)** |
| Visual | 318 | 12.8 (7.5) | 82 (25.8) | 1.13 (0.85-1.49) | 1.19 (0.90-1.59) |
| Sensory | 539 | 12.9 (7.0) | 123 (22.8) | 1.0 (reference) | 1.0 (reference) |
| EDSS 6 | | | | | |
| Motor | 194 | 14.8 (7.4) | 53 (27.3) | **1.75 (1.24-2.47)** | **1.67 (1.17-2.36)** |
| Brainstem/cerebellar | 334 | 13.8 (7.2) | 12 (12.0) | 1.29 (0.93-1.79) | 1.18 (0.85-1.65) |
| Visual | 318 | 14.6 (7.3) | 47 (14.8) | 0.97 (0.68-1.39) | 1.05 (0.73-1.51) |
| Sensory | 539 | 14.8 (7.0) | 83 (15.4) | 1.0 (reference) | 1.0 (reference) |

^1^unadjusted; ^2^adjusted for age, sex, ancestry, calendar year of disease onset, educational attainment, smoking, BMI status, and DMT exposure. Individuals who had reached an outcome at diagnosis were excluded.

eTable 6. Demographic and lifestyle factors associated with onset symptom type (hierarchical classification; reference: sensory). Age at diagnosis >25 years.

| Motor onset (n=157) | | |
| --- | --- | --- |
| Predictor | OR (95% CI) | OR (95% CI)^1^ |
| Male | **1.56 (1.03-2.27)** |  |
| Non-Nordic | **1.64 (1.06-2.53)** |  |
| Pre-secondary education Post-secondary education | 0.79 (0.89-3.19) 1.09 (0.73-1.63) |  |
| >Median age at onset | 1.38 (0.94-2.03) | 1.01 (0.99-1.04) |
| 1-10 pack years of smoking >10 pack years of smoking | 1.10 (0.70-1.71) **1.57 (1.00-2.63)** | **1.05 (1.00-1.11)** |
| BMI | 0.96 (0.51-1.79) | 0.99 (0.98-1.03) |
| Brainstem/cerebellar (n=276) | | |
| Male | 1.38 (0.99-1.93) |  |
| Non-Nordic | 1.38 (0.94-2.02) |  |
| Pre-secondary education Post-secondary education | 1.32 (0.70-2.49) 1.07 (0.77-1.50) |  |
| Higher education | 0.87 (0.67-1.13) |  |
| Age at onset | 1.15 (0.84-1.57) | 1.02 (1.00-1.04) |
| 1-10 pack years of smoking >10 pack years of smoking | 1.52 (0.93-1.85) **1.73 (1.14-2.63)** | **1.03 (1.01-1.05)** |
| BMI | 1.30 (0.80-2.12) | 1.00 (0.97-1.04) |
| Visual (n=257) | | |
| Male | 0.78 (0.54-1.12) |  |
| Non-Nordic | 1.24 (0.83-1.88) |  |
| Pre-secondary education Post-secondary education | 1.07 (0.51-2.25) 1.44 (0.94-2.02) |  |
| Higher education | 0.81 (0.62-1.06) |  |
| Age at onset | 0.84 (0.61-1.15) | **0.98 (0.96-0.99)** |
| 1-10 pack years of smoking >10 pack years of smoking | 1.14 (0.81-1.59) 1.15 (0.72-1.84) | 1.00 (0.99-1.04) |
| BMI | **2.05 (1.24-3.39)** | **1.02 (1.00-1.04)** |

Sensory onset, co-occurring only with cognitive or autonomic domains, was used as the reference category (n=453). All variables were entered simultaneously in the model. ^1^For continuous predictors, estimates correspond to a 1-unit increase (years, pack-years of smoking, BMI).
